# Of Timescales, Animal Models, and Human Disease: The 50th Anniversary of *C. elegans* as a Biological Model

**DOI:** 10.3389/fneur.2013.00129

**Published:** 2013-10-01

**Authors:** Denes V. Agoston

**Affiliations:** ^1^Department of Anatomy, Physiology and Genetics, Uniformed Services University, Bethesda, MD, USA

**Keywords:** disease models, animal, translational research, trauma, nervous system, inflammation, clinical studies

In 2007, the Faculty Senate at the Uniformed Services University (USU) in Bethesda, MD, USA, invited Dr. Sydney Brenner, one of the creative geniuses of modern genetics, to deliver the annual David Packard Lecture. “No powerpoint or any visual aid necessary; one good phrase is worth a thousand slides,” Dr. Brenner said before his presentation – and he was right. He delivered a brilliant talk entitled *Humanities’ Genes*. One of the main points of his lecture was that “the next model system is ourselves,” which could have been the subtitle. Dr. Brenner presented compelling arguments (using his own disease condition also as an example) of why in the twenty-first century biomedical scientists can (and should) switch from animal modeling of human diseases to studying the real subject: man. This may come as a surprise from one of the founders of molecular biology. Dr. Brenner's early work includes the discovery of messenger and transfer RNA, frameshift mutations, the triplet nature of the genetic code, and the creation of the first computer matrix analysis of nucleic acids.

In 1963, exactly 50 years ago, Dr. Brenner wrote to Dr. Max Perutz^1^: “it is now widely realized that nearly all the ‘classical’ problems of molecular biology have either been solved or will be solved in the next decade. Because of this, I have long felt that the future of molecular biology lies in the extension of research to other fields of biology, notably development and the nervous system.” Then he described his vision for future studies: “we should like to attack the problem of cellular development in a similar fashion, choosing the simplest possible differentiated organism and subjecting it to the analytical methods of microbial genetics. Thus we want a multicellular organism which has a short life cycle, can be easily cultivated, and is small enough to be handled in large numbers, like a micro-organism. It should have relatively few cells, so that exhaustive studies of lineage and patterns can be made, and should be amenable to genetic analysis.”

Accordingly, Dr. Brenner introduced *Caenorhabditis briggsae* (a close relative of *C. elegans*) – a non-parasitic nematode – as a model organism to study complex biological regulations at the molecular level. Seminal discoveries made using *C. elegans* as a model include cell fate mapping, the genetics of basic nervous system development, cell fate determination, programed death (apoptosis), RNA interference, and more. *C. elegans* has served biology extremely well during the last 50 years as reflected in six Nobel Prizes using the model – one of which Dr. Brenner shared with Drs. Horvitz and Sulston in Physiology or Medicine for their discoveries about the genetic regulation of organ development and programed cell death (1).

In his lecture at USU, Dr. Brenner argued that biology has reached another inflection point: rodent models may be close to the end of their usefulness as far as human disease modeling is concerned. Our understanding of basic biological processes has grown immensely thanks to studies utilizing *C. elegans*, other “simple” organisms, and rodent models. However, human diseases are far too complex and can therefore only be poorly mimicked in their entirety in rodents. Importantly, he indicated that the state of genetics, systems biology, and informatics – I would also add the various *in vivo* imaging techniques – enable biomedical scientists to study human diseases in a non-invasive fashion like never before in the new “model system” (man).

Using rodent models myself to study neuroinflammation and other pathologies following mild blast-induced traumatic brain injury (TBI) (2), Dr. Brenner's message came to me as I was reading the paper “Genomic responses in mouse models poorly mimic human inflammatory disease” (3). The work by Seok, Warren, and Cuenca (and their 30 plus co-authors with contributions by the Inflammation and Host Response to Injury, Large Scale Collaborative Research Program) compared the genomic (transcriptional) responses to burn, trauma, and endotoxemia in humans and their respective mouse models. The authors have shown that the three different conditions trigger strikingly similar inflammatory responses in humans (detected at the level of the transcriptome) regardless of the etiology (i.e., burn, trauma, or endotoxemia). However, the transcriptional responses detected in the mouse models were strikingly different from their human counterparts. Moreover, the transcriptional responses among the three different mouse models were dissimilar.

The authors’ work also shows that the significantly regulated pathways in the human diseases are distinct from their respective murine models. Finally – and very importantly – the authors demonstrated that the temporal pattern of changes (“gene response time” or “recovery times to normal values”) in the human conditions were substantially different from their corresponding murine models. While gene response times were similar between rodents and humans (6–12 h) for all three conditions, recovery times were on entirely different scales: weeks to months (even a year) in humans and less than 10 days in their respective murine models. For instance, recovery time for burns in mice was 7 days whereas in humans it was over one full year. While there are other potential explanations for the observed discrepancies (some of which are discussed online)^2^^,^^3^, these observations highlight a very critical yet almost completely neglected aspect of animal modeling: the different timescales.

Biological processes run on vastly different timescales in humans compared to the various animal models used in biomedical research. After all, the fast lifecycle of *C. elegans* (embryogenesis ∼12 h, development to the adult stage occurs in 2.5 days, and the life span is 2–3 weeks) was one of the key features that the worm was selected as a model organism. Compare this with the gestational period in mice (19–21 days) and humans (∼266 days) – to focus on a relatively well-conserved, basic biological process between rodents and humans. Timescale differences in other fundamental biological processes (e.g., sexual maturity, lifespan, and metabolism) in rodents versus humans illustrate rather well that a “rat day” is not equivalent to a human day; and studies comparing the temporal patterns of complex, molecular, and system level changes between the two species are limited. Consequently, we currently lack inter-species “conversion factor(s)” (or rather an algorithm) that would enable comparisons and adjustments between the temporal patterns of basic physiological processes as well as pathological mechanisms (e.g., inflammation).

The approach taken by the authors of the PNAS paper (comparing rodent and human genomic responses) maybe one potential approach to developing such a temporal conversion factor between some rodent models of induced human pathologies (e.g., burns, TBI) where the time of the insult is known. In the case of burns, obtaining biosamples from rodents within minutes after the insult, continuing to sample at a high temporal resolution for up to 7–10 days (the recovery time for rodents as indicated by the authors), and comparing all rodent samples to samples obtained from human patients at the same post-injury time points but collected for up to 1 year (the recovery time for humans) can identify similarities (if any) between patterns of genomic inflammatory responses in the two different organisms.

Establishing “conversion factor(s)” (or algorithm) between rodent and human time scales for inflammatory processes (among others) would be critical due to inflammation's key role in various diseases including TBI. Given the lack of efficacious drugs for TBI (4), the field would benefit from (re)designing clinical trials using such evidence-based timescale for testing anti-inflammatory treatments that have worked in rodents (e.g., minocycline). Such a conversion factor/algorithm would also enable us to better compare outcomes between animal models and the clinical population in TBI (5).

In summary, we biomedical scientists – especially those of us performing “translational” research – need to pay closer attention to the differences between human and rodent timescales in order to draw more clinically useful conclusions from our experimental work. And whenever methodologically and ethically possible, we may want to seriously consider Dr. Brenner's advice: “the next (and maybe best) model system is ourselves.”

## Footnotes

^1^http://elegans.som.vcu.edu/Sydney.html

^2^http://www.pnas.org/content/early/2013/07/11/1308333110.full.pdf+html

^3^http://www.pnas.org/content/early/2013/07/10/1307452110.full.pdf?etoc

## References

[1] Brenner S Nobel lecture. Nature's gift to science. Biosci Rep (2003) 23:225–37 10.1023/B:BIRE.0000019186.48208.f3 15074543

[2] Kovesdi E Kamnaksh A Wingo D Ahmed F Grunberg NE Long JB Acute minocycline treatment mitigates the symptoms of mild blast-induced traumatic brain injury. Front Neurol (2012) 3:111 10.3389/fneur.2012.00111 22811676 PMC3397312

[3] Seok J Warren HS Cuenca AG Mindrinos MN Baker HV Xu W Genomic responses in mouse models poorly mimic human inflammatory diseases. Proc Natl Acad Sci U S A (2013) 110:3507–12 10.1073/pnas.1222878110 23401516 PMC3587220

[4] Agoston DV Risling M Where will the (new) drugs for traumatic brain injury treatment be coming from? Front Neurol (2012) 3:27 10.3389/fneur.2012.00107 22403571 PMC3291867

[5] Agoston DV Risling M Bellander BM Bench-to-bedside and bedside back to the bench; coordinating clinical and experimental traumatic brain injury studies. Front Neurol (2012) 3:3 10.3389/fneur.2012.00003 22347208 PMC3270391

